# The Occurrence and Risk Factors of Black Triangles Between Central Incisors After Orthodontic Treatment

**DOI:** 10.3390/diagnostics14232747

**Published:** 2024-12-06

**Authors:** Ji-Song Jung, Ho-Kyung Lim, You-Sun Lee, Seok-Ki Jung

**Affiliations:** 1 Department of Orthodontics, Graduate School of Clinical Dentistry, Korea University, Seoul 02841, Republic of Korea; maerypaw@naver.com; 2 Department of Oral and Maxillofacial Surgery, Korea University Guro Hospital, Seoul 08308, Republic of Korea; ungassi@korea.ac.kr; 3 Department of Orthodontics, Korea University Anam Hospital, Seoul 02841, Republic of Korea; 4 Department of Orthodontics, Korea University Guro Hospital, Seoul 08308, Republic of Korea

**Keywords:** black triangle, open gingival embrasure, orthodontic treatment

## Abstract

Background/Objectives: To assess the incidence of and risk factors for black triangles between the central incisors after orthodontic treatment; Methods: Ninety-seven post-treatment patients (29 men and 68 women; mean age, 22.7 years) were retrospectively divided into two groups based on the presence or absence of black triangles, using intraoral photographs. Based on the Jemt Index, the black triangle occurrence group was further classified into mild, moderate, and severe groups. Parameters from periapical radio graphs, lateral cephalograms, and study models were compared between the occurrence and the non-occurrence groups by using independent *t*-tests. Logistic regression analysis was performed to identify the risk factors for black triangles; Results: The incidence of black triangles between the central incisors was 51% and 64% in the maxilla and in the mandible, respectively. The factors significantly associated with the occurrence of black triangles were age, treatment duration, the lingual inclination of the maxillary incisors in the maxilla, and age in the mandible (*p* < 0.05); Conclusions: This study showed the diverse risk factors associated with black triangles between central incisors after orthodontic treatment and revealed that the formation of black triangles is relatively common. Considering these risk factors during orthodontic diagnosis and treatment planning can help minimize the occurrence of black triangles.

## 1. Introduction

Over the past few decades, the goals of patients seeking orthodontic treatment have continuously evolved. In the 1980s and 1990s, patients primarily aimed to resolve malocclusion or restore normal occlusal function through orthodontic treatment. However, recent patients place a significant emphasis not only on functional occlusal recovery but also on aesthetic improvement [1,2,3].

Orthodontic treatment can induce unwanted side effects on periodontal tissues, such as root resorption, bone dehiscence, gingival recession, and the formation of black triangles, which may reduce patient satisfaction with the treatment [2,4,5]. A black triangle is an empty space beneath the contact area between teeth due to the loss of the interdental papilla [6]. This issue not only affects aesthetics but can also lead to periodontal problems by chronically trapping food debris and making plaque control more difficult. Therefore, a thorough understanding of the etiology and pathogenesis of black triangles, as well as appropriate orthodontic diagnosis and treatment, is required [7].

Previous studies have identified various factors contributing to the development of black triangles, including patient age, history of periodontitis, the distance between the interproximal contact and the alveolar crest, gingival biotype, and morphological characteristics of teeth [8]. However, there is still a lack of consensus regarding the relationship between orthodontic treatment and black triangles. Some studies have reported a high incidence of black triangles in patients who have undergone orthodontic treatment [9,10,11,12,13,14,15,16], while others have suggested that orthodontic treatment can stimulate the formation of interdental papillae, thus reducing the incidence of black triangles [17,18,19]. Consequently, a definitive conclusion about the relationship between orthodontic treatment and black triangles has yet to be reached. In addition, it should be distinguished from the case where black triangles occur when orthodontic treatment is performed on normal periodontal tissue with reduced periodontal support.

Risk factors associated with black triangle formation after orthodontic treatment include age, treatment duration, tooth shape, the amount of tooth movement, root angulation after treatment, the degree of crowding, the distance between the alveolar crest and the interproximal contact after treatment, traumatic brushing, the occurrence of periodontitis, and gingival biotype [20,21,22,23,24,25,26]. However, most previous studies have evaluated the impact of risk factors on black triangle formation in a unidimensional manner, which limits the understanding of how these factors might interact during orthodontic treatment. Therefore, comprehensive investigation of associations among various risk factors is necessary.

The purpose of this study was to examine the association between the occurrence of black triangles during orthodontic treatment and factors such as age, gender, bracket type, and extraction status. The null hypothesis of this study is that there is no correlation between the occurrence of black triangles during orthodontic treatment and factors such as age, gender, bracket type, and extraction status.

## 2. Materials and Methods

### 2.1. Subjects

Patients who completed orthodontic treatment at the Department of Orthodontics, Korea University Guro Hospital were selected for this study. Among them, patients without a black triangle between the maxillary and mandibular central incisors, as assessed by a Jemt Index score of 3 from intraoral photographs taken before orthodontic treatment, were included. Patients with periodontitis, anterior tooth loss, a history of previous orthodontic treatment, prosthetic restorations of the maxillary or mandibular central incisors, or those who underwent interproximal reduction (stripping) during orthodontic treatment were excluded from the study. A total of 300 patients who underwent orthodontic treatment between January 2020 and March 2022 were evaluated, and after applying the exclusion criteria, 97 patients (58 adults and 39 adolescents, 47 extraction and 50 non-extraction) who had pre- and post-treatment intraoral photographs, lateral cephalograms, periapical radiographs, and study models were selected for this retrospective study.

For age classification, adolescents were defined as patients aged 9 to under 20 years, and adults were defined as those aged 20 to under 55 years based on pre-treatment age. The extraction group consisted of patients who underwent the extraction of the first premolars in both the maxilla and mandible. The orthodontic appliances used for treatment were the 0.022-inch slot metal ligating brackets (Formula-R, Tomy Inc, Fukushima, Japan), self-ligating brackets (Clippy-C, Tomy Inc, Fukushima, Japan), and lingual brackets (Fujita, Succeeding Co, Morioka, Japan).

### 2.2. Measurement Methods

#### 2.2.1. Classification of Black Triangles According to Severity

**Figure 1:**
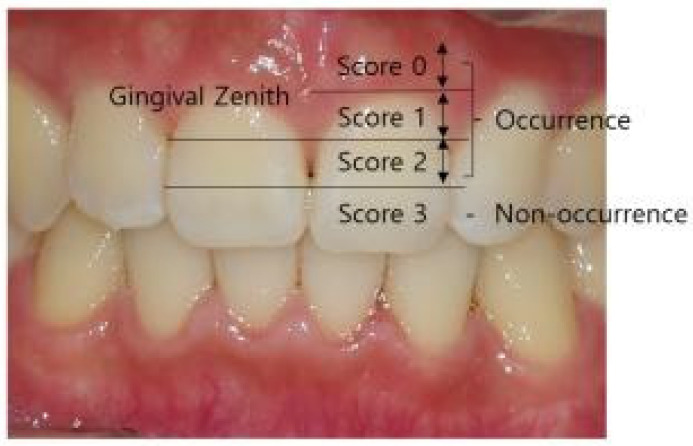
Classification of black triangles according to Jemt Index.

Intraoral frontal photographs of the maxillary and mandibular central incisors were taken within one week after the completion of orthodontic treatment. Intraoral photographs were taken from the front using standardized camera settings (shutter speed 1/125, ISO 200, F22) after drying the area with air. A parallel line was drawn from the gingival zenith (the most cervical point of the crown) and the most cervical point of the interproximal contact area, followed by a bisecting line through the midpoint of these two parallel lines, dividing the interdental papilla area into four sections. Based on the Jemt Index, patients were classified into two groups: the non-occurrence group (Score 3, the papilla fills up the entire proximal space) and the occurrence group, which was further subdivided into Score 2 (mild, half or more of the height of the papilla is present), Score 1 (moderate, less than half of the height of the papilla is present), and Score 0 (severe, no papilla is present) (Figure 1).

#### 2.2.2. Measurements of Tooth Movement on Lateral Cephalograms

The angular and anteroposterior, as well as vertical, changes in the maxillary and mandibular central incisors before and after orthodontic treatment were measured using lateral cephalometric radiographs. Each radiograph was taken in a natural head position. Additionally, all subjects were instructed to stand still, hold their breath, swallow briefly before imaging, and refrain from swallowing while the radiographs were being taken. For the maxillary central incisors, the angular change was assessed by measuring the difference in the angle between the upper incisor and the SN plane (ΔU1 to SN), and for the mandibular central incisors, the difference in the incisor mandibular plane angle (ΔIMPA) was measured relative to the mandibular plane before and after orthodontic treatment.

**Figure 2:**
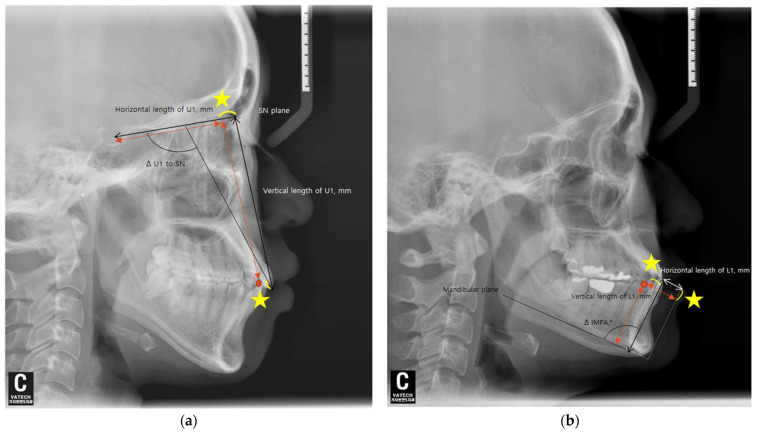
Lateral cephalograms show measurements of tooth movement. (**a**) SN, Sella–Nasion; U1, the maxillary central incisor; Δ U1 to SN refer to the changes in measurements between before (T1) and after (T2) treatment (Δ = T2 − T1). (**b**) L1, the mandibular central incisor; IMPA, incisor mandibular plane angle. Δ IMPA refers to the changes in measurements between before (T1) and after (T2) treatment (Δ = T2 − T1).

The horizontal movement of the maxillary central incisors was measured as calculating the change in the distance between the perpendicular line drawn from the tip of the maxillary central incisor crown to the SN plane and the distance from this point to the Sella (S) (ΔTH = TH2 − TH1). The vertical movement of the maxillary central incisors was calculated by measuring the change in the perpendicular distance from the tip of the maxillary central incisor crown to the SN plane (ΔTV = TV2 − TV1) (Figure 2a).

For the mandibular central incisors, the horizontal movement was assessed by measuring the change in the straight-line distance between the tip of the mandibular central incisor crown and the perpendicular line drawn from the mandibular plane to the Pogonion (ΔTH = TH2 − TH1). The vertical movement was calculated by measuring the change in the perpendicular distance from the tip of the mandibular central incisor crown to the mandibular plane (ΔTV = TV2 − TV1) (Figure 2b).

#### 2.2.3. The Tooth Shape and the Height of Alveolar Bone

In periapical radiographs taken before orthodontic treatment, the long axis of the maxillary and mandibular central incisors was established. The ratio of the perpendicular distance from the long axis to the mesial contact point of the crown (1) to the perpendicular distance from the long axis to the mesial CEJ (cemento–enamel junction) (2) was calculated (crown ratio = (1)/(2)) [27].

**Figure 3:**
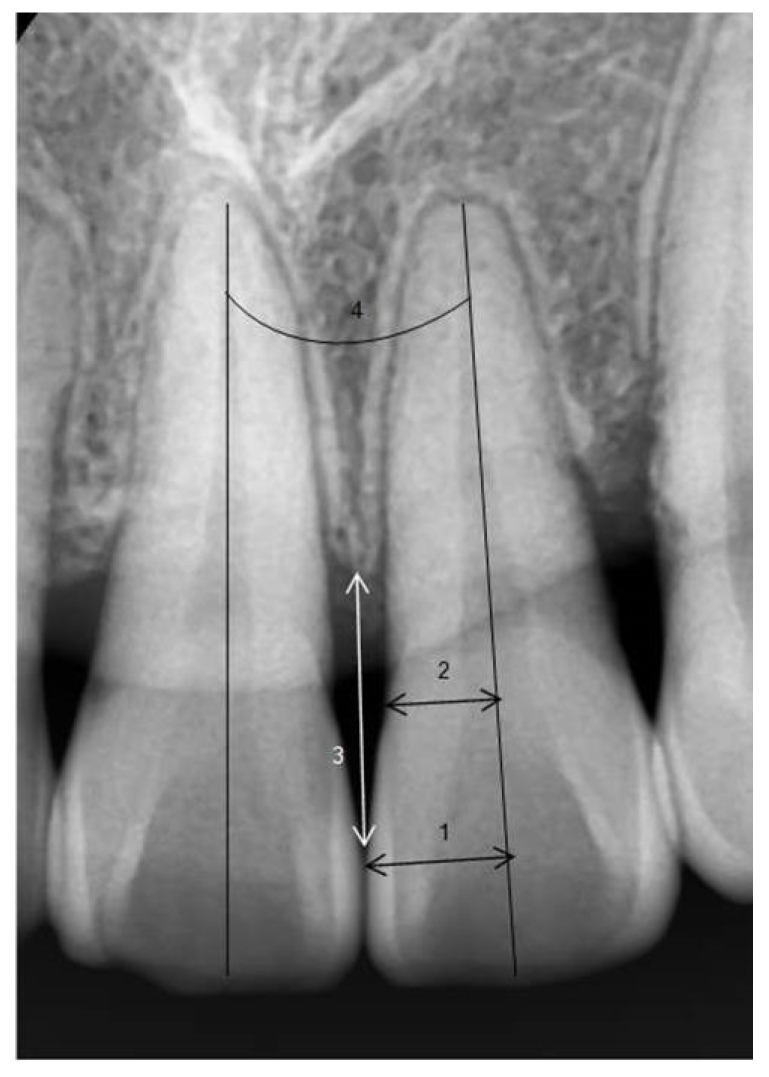
Periapical radiographic measurements. 1, perpendicular distance from the mesial interproximal contact to the tooth long axis; 2, perpendicular distance from the mesial CEJ to the tooth long axis; 3, distance of alveolar bone–interproximal contact; 4, post-treatment root angulation classification of black triangle according to Jemt Index.

To minimize errors due to magnification or distortion in periapical radiographs, the clinical average crown lengths of the central incisors were used as reference values, and the amount of alveolar bone height change (ΔTA = TA2 − TA1) was measured [28]. The distance from the contact point (or the most cervical point of the contact surface) of the maxillary and mandibular central incisors to the alveolar crest was measured, keeping the measurement parallel to the long axis of the left central incisor, and the difference in distance before and after orthodontic treatment was compared. If there was a space between the teeth without a contact point, the distance from the narrowest point between the mesial surfaces of both crowns and the alveolar crest was measured. The angle between the roots of the left and right maxillary and mandibular central incisors was measured in patients after completing orthodontic treatment (Figure 3).

#### 2.2.4. Measurements of Crowding on Study Models

**Figure 4:**
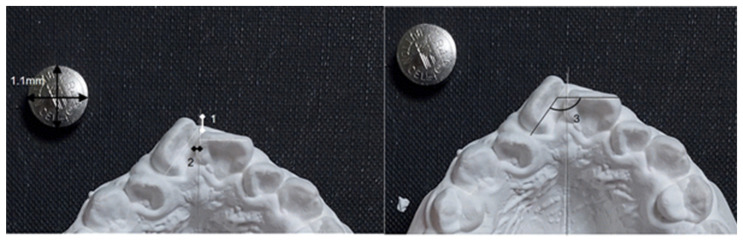
Measurements of the anteroposterior and transverse overlap of the two central incisors and the angle formed by the incisal edges. 1, degree of vertical overlap; 2, degree of horizontal overlap; 3, amount of crown rotation.

In pre-treatment study model photographs, the anteroposterior and mesiodistal crowding of the maxillary and mandibular central incisors was measured. The plane connecting the central incisors and the first molars was set as the occlusal plane, and the photographs were taken perpendicular to this occlusal plane. The midpalatal raphe in the maxilla and a perpendicular line bisecting the line connecting the mesial surfaces of the first molars in the mandible were used as the reference lines. Anteroposterior overlap was measured by the length of the line connecting the most mesial points of the left and right central incisors, drawn parallel to the reference line. Mesiodistal overlap was measured by the length of the perpendicular line drawn from the most mesial points of the left and right central incisors to the reference line. The angle change between the crowns of the left and right central incisors (ΔTR = TR2 − TR1) was measured using occlusal surface photographs of the study models before and after orthodontic treatment. (Figure 4).

### 2.3. Statistical Analysis

Statistical analysis was performed using PASW Statistics version 18.0.0 (SPSS Inc., Chicago, IL, USA). The target sample size was determined based on a review of existing literature and calculations using the G Power program version 3.1.9.7. To confirm the association between the incidence rates and contributing factors in the two groups, the statistically significant minimum sample size was calculated to be 40 participants per group based on age, requiring a total of at least 80 participants. All measurements were conducted by a single examiner, who randomly selected and reanalyzed 25 patients at one-week intervals. The degree of black triangle occurrence after orthodontic treatment was assessed, and the association between black triangle occurrence and variables such as age, gender, bracket type, and extraction versus non-extraction groups was examined using the Chi-square test and Fisher’s exact test.

For the occurrence and non-occurrence groups of black triangles, mean values and standard deviations were analyzed and compared using the independent *t*-test for variables such as treatment duration, age, tooth shape, pre- and post-treatment angle differences of the maxillary and mandibular incisors, horizontal and vertical movement changes of the maxillary and mandibular incisors, pre- and post-treatment differences in alveolar bone height, post-treatment angle between the roots of the left and right central incisors, anteroposterior and mesiodistal crowding of the maxillary and mandibular central incisors before treatment, and crown rotation changes before and after treatment.

Additionally, the independent *t*-test was performed to compare mean values for age (pediatric/adolescent versus adult) and extraction status (extraction versus non-extraction), which were identified as risk factors associated with black triangle occurrence in the Chi-square test. Simple logistic regression analysis was conducted to assess the association between risk factors and the occurrence of black triangles in the maxillary and mandibular central incisors during orthodontic treatment, followed by multiple logistic regression analysis to further analyze the combined risk factors and calculate the odds ratios. Statistical significance was set at a *p*-value of less than 0.05.

## 3. Results

### 3.1. Association Between Black Triangle Occurrence and Each Group

The number distribution of subjects in each group is presented in Table 1. The intraclass correlation coefficient (ICC) was used to determine the intra-examiner reliability of the measurements, which were scored as follows: ICC < 0.4, poor reliability; 0.4 < ICC < 0.75, moderate reliability; ICC > 0.75, excellent reliability. The ICC values in this study ranged from 0.93 to 0.99, demonstrating excellent reliability. Among the 97 patients, black triangles occurred in 51% of the maxillary incisors and 64% of the mandibular incisors after orthodontic treatment (Table 2). When comparing the occurrence of black triangles between adolescents and adults, adolescents showed a black triangle occurrence rate of 17.9% in the maxilla and 38% in the mandible, while adults had rates of 74% in the maxilla and 82% in the mandible, indicating a significant association between age and black triangle occurrence (Table 3). Among the extraction and non-extraction groups, only the maxilla in the extraction group showed a significant association with black triangle occurrence, while the type of bracket used did not show a significant difference in black triangle formation (Table 4 and Table 5).

### 3.2. Comparison of Mean Values Between the Black Triangle Occurrence and Non-Occurrence Groups

When comparing the mean values between the black triangle occurrence and non-occurrence groups using the independent *t*-test, significant differences were observed in the maxilla for treatment duration, age, and the angle change of the central incisors before and after orthodontic treatment, and in the mandible for age. In the maxilla, the treatment duration was significantly longer in the occurrence group (40.00 ± 19.21 months) than in the non-occurrence group (29.96 ± 11.48 months). Additionally, the mean age in the occurrence group (28.14 ± 10.88 years) was significantly higher than that in the non-occurrence group (16.94 ± 5.92 years). In terms of the angle change of the maxillary central incisors relative to the SN plane, the occurrence group showed more lingual inclination (9.18 ± 1.10°) compared to the non-occurrence group (2.12 ± 1.29°) (Table 6).

When comparing the mean values between adolescents and adults in the maxilla using the independent *t*-test, adults showed a relatively longer treatment duration and greater lingual inclination of the maxillary central incisors (Table 7). Additionally, when comparing the extraction and non-extraction groups, the extraction group exhibited greater lingual inclination of the maxillary central incisors (Table 8).

### 3.3. Association Between Black Triangles and Risk Factors

The association between expected risk factors and the occurrence of black triangles was examined using simple regression analysis. In the maxilla, treatment duration, age, and the angle change of the maxillary central incisors before and after orthodontic treatment were identified as significant risk factors, while in the mandible, age was found to be a significant risk factor. Multiple regression analysis was then conducted to reanalyze these risk factors collectively. The results indicated that age and the angle change of the central incisors before and after orthodontic treatment were significant contributing factors in the maxilla, while age remained a significant factor in the mandible. In the maxilla, age showed an odds ratio of 1.087, and the angle change of the central incisors showed an odds ratio of 0.940. In the mandible, age had an odds ratio of 1.197 (Table 9 and Table 10).

## 4. Discussion

In this study, the incidence of black triangles after orthodontic treatment was found to be between 51% and 64%. This is higher than the previously reported rates of 38% to 58%, which can be attributed to differences in study design, sample size, age distribution, and methods for assessing black triangles [7,9,29]. The study confirmed a significant association between the occurrence of black triangles during orthodontic treatment and factors such as age and extraction status. Previous studies that highlighted the advantages of non-extraction treatment have reported that post-extraction orthodontic treatment leads to a significant reduction in the width of keratinized gingiva in the anterior region compared to non-extraction patients [2]. This suggests that extraction treatment may have a negative impact on periodontal tissues. However, as with other studies analyzing the effects of bracket type and bonding location on microbial distribution and periodontal health [30,31,32], this study found no significant association between the type of brackets or their bonding location and the occurrence of black triangles.

When comparing the mean values of various risk factors between patients with and without black triangles, the study found significant differences in the maxilla regarding treatment duration, age, and pre- and post-treatment incisor angulation changes. In the mandible, only age showed a significant difference. Previous studies have reported a significant increase in periodontal disease risk, such as inflammatory gingival hyperplasia, with longer orthodontic treatment durations due to increased plaque accumulation on appliances like brackets and power chains, as well as poor oral hygiene habits [33,34]. However, Ko-Kimura et al. [22] reported no significant statistical correlation between the duration of orthodontic treatment and the occurrence of black triangles. In this study, the occurrence of black triangles in the maxillary anterior region was significantly higher in patients with longer treatment durations than those without black triangles.

The direction of tooth movement has also been identified in previous studies as a risk factor for the formation of black triangles during orthodontic treatment. Specifically, numerous studies have reported that labial horizontal or labial angular movements of the maxillary anterior teeth increase the likelihood of gingival recession or black triangle formation due to thin labial alveolar bone [35,36]. In this study, of the 97 patients, only 27 exhibited labial angular movement during orthodontic treatment, and only 8 of these patients developed mild black triangles, scoring 2 on the Jemt Index. The remaining 19 patients did not develop black triangles. Therefore, no significant association was found between labial angular movement and black triangle formation, contrary to previous studies. However, the results of the multiple regression analysis showed that the likelihood of black triangle formation increased as the maxillary incisors underwent lingual angulation relative to the SN plane. The odds ratio for changes in maxillary incisor angulation was 0.940, indicating that for each degree of lingual angulation, the risk of black triangle formation increased by a factor of 1.06. This finding aligns with An et al. [7], who reported a significant association between black triangle formation and lingual horizontal movement of the maxillary incisors during orthodontic treatment.

The multiple regression analysis also revealed a significant association between age and the occurrence of black triangles during orthodontic treatment in both the maxilla and mandible. The odds ratios were 1.087 for the maxilla and 1.197 for the mandible, indicating that for each year of age, the risk of black triangle formation increased by 1.087 times in the maxilla and 1.197 times in the mandible. While age is commonly associated with an increased risk of periodontal disease, the occurrence of black triangles in older patients is not solely due to poor oral hygiene over time. As aging progresses, the reduction in oral epithelial keratinization, decreased interdental papilla height, and increased interdental space contribute to gingival recession and a higher likelihood of black triangles in adults [22,24]. These effects are particularly pronounced in adults over the age of 20 who undergo orthodontic treatment, as the ability of the papilla to regenerate and fill interdental spaces appears to diminish with age, exacerbating the condition during orthodontic interventions.

Tarnow et al. [21] reported a correlation between the distance from the contact point to the alveolar crest and the occurrence of black triangles. When the distance exceeds 5 mm, the interdental papilla cannot fill the black triangle, but when the distance is less than 5 mm, the papilla can re-fill the space. This study measured only the relative distance changes between the contact point and the alveolar crest before and after orthodontic treatment, and no significant association with black triangle formation was found. However, it was observed that alveolar bone height generally decreased in patients, regardless of whether black triangles developed. This suggests that age-related changes, typically associated with aging, were evident during the course of orthodontic treatment.

Future studies should classify patients according to the type and severity of malocclusion and their ability to maintain oral hygiene to investigate black triangle occurrence rates. Additionally, selecting a control group of patients who did not undergo orthodontic treatment for comparative analysis would be beneficial. The relationship between black triangle formation and gingival biotype should also be examined, as gingival phenotype plays a crucial role in determining susceptibility to black triangles. Thin gingival phenotypes are particularly prone to gingival recession and papilla loss, which can exacerbate black triangle formation. Evaluating these factors in future studies could enhance clinical decision-making. Furthermore, while this study did not directly compare fixed and removable appliances, future investigations should explore their respective impacts on black triangle formation to develop clearer guidelines for appliance selection. The risk factors associated with black triangles after orthodontic treatment are diverse. Being aware of these factors and predicting potential risks during diagnosis and treatment planning can help prevent or minimize the occurrence of black triangles.

Once black triangles form, various approaches can be considered for correction depending on the severity and underlying cause. Orthodontic techniques, such as controlled tipping or closing interdental spaces, can help reduce black triangle visibility, while interproximal stripping can minimize interdental spacing by reducing tooth size. Restorative approaches, including composite resins or veneers, may also be employed to address aesthetic concerns. Interdisciplinary collaboration between orthodontics, periodontics, and prosthodontics is necessary to reduce or eliminate black triangles that develop after orthodontic treatment.

## 5. Conclusions

This study showed that the occurrence of black triangles was significantly associated with age and extraction status, with a higher incidence observed in adults compared to adolescents and in extraction cases compared to non-extraction cases. In the maxilla, age, changes in incisor angulation relative to the SN plane, and treatment duration showed significant associations with the occurrence of black triangles after orthodontic treatment. In the mandible, only age was significantly associated.

## Figures and Tables

**Table 1:** Number distribution of subjects with each group.

Group		Subjects, *n* (%)
Gender	Male	29 (30%)
	Female	68 (70%)
Age	Young	39 (40%)
	Adult	58 (60%)
Extraction	Extraction	47 (48%)
	Non-Extraction	50 (52%)
Brackets	Ligating	51 (53%)
	Self-ligating	36 (37%)
	Lingual	10 (10%)

**Table 2:** Incidence of black triangle (*n* = 97).

	Non-Occurrence	Occurrence		
		Mild	Moderate	Severe
Maxilla	47 (48%)	49 (51%)	1 (1%)	0 (0%)
Mandible	34 (35%)	59 (61%)	4 (4%)	0 (0%)

**Table 3:** Comparison of black triangle incidence between young and adult in the occurrence group ^†^.

	Young	Adult	Significance
Maxilla	7 (14%)	43 (86%)	***
Mandible	15 (24%)	48 (76%)	***

^†^ Chi-square test was used to compare the incidence between young and adult. * *p* < 0.05; ** *p* < 0.01; *** *p* < 0.001.

**Table 4:** Comparison of black triangle incidence between extraction and non-extraction in the occurrence group ^†^.

	Extraction	Non-extraction	Significance
Maxilla	32 (64%)	18 (36%)	**
Mandible	32 (51%)	31 (49%)	NS

^†^ Chi-square test was used to compare the incidence between extraction and non-extraction. NS, not significant; * *p* < 0.05; ** *p* < 0.01; *** *p* < 0.001.

**Table 5:** Comparison of black triangle incidence between bracket types in the occurrence group ^†^.

	Ligating	Self-Ligating	Lingual	Significance
Maxilla	24 (48%)	18 (36%)	8 (16%)	NS
Mandible	29 (46%)	26 (41%)	8 (13%)	NS

^†^ Chi-square test was used to compare the incidence between bracket types. NS, not significant; * *p* < 0.05; ** *p* < 0.01; *** *p* < 0.001.

**Table 6:** Comparison of black triangle incidence between the occurrence and non-occurrence groups in the maxilla and mandible (means ± SD) ^†^.

	Maxilla			Mandible		
	Occurrence	Non-Occurrence	Significance	Occurrence	Non-Occurrence	Significance
Treatment period, month	40.00 ± 19.21	9.96 ± 11.48	*	37.29 ± 17.42	31.15 ± 14.39	NS
Age, y	28.14 ± 10.88	16.94 ± 5.92	***	26.21 ± 10.65	16.24 ± 6.10	***
Tooth shape	1.24 ± 0.12	1.48 ± 1.90	NS	1.27 ± 0.14	1.27 ± 0.11	NS
ΔU1 to SN /ΔIMPA, °	−9.18 ± 1.10	−2.12 ± 1.29	***	2.19 ± 1.17	3.96 ± 2.86	NS
Horizontal movement of U1/L1, mm	−0.45 ± 0.66	−0.33 ± 1.05	NS	−0.11 ± 2.02	−0.37 ± 1.40	NS
Vertical movement of U1/L1, mm	1.64 ± 12.87	1.62 ± 13.33	NS	1.82 ± 13.08	−0.06 ± 1.00	NS
Δ Distance from ICP to ABC, mm	−0.64 ± 0.76	−0.50 ± 0.30	NS	−0.86 ± 1.28	0.46 ± 0.24	NS
Root angulation at T2, °	4.03 ± 1.69	4.04 ± 2.03	NS	3.81 ± 2.44	5.04 ± 1.72	NS
A-P overlapped distance, mm	0.10 ± 0.20	0.04 ± 0.01	NS	0.09 ± 0.28	0.61 ± 0.17	NS
Transverse overlapped distance, mm	−0.51 ± 0.23	−0.02 ± 0.17	NS	0.01 ± 0.04	−0.00 ± 0.04	NS
Rotation, °	11.49 ± 9.60	13.57 ± 6.10	NS	13.08 ± 9.84	11.93 ± 3.55	NS

^†^ B Independent *t*-tests were used to compare between the occurrence and non-occurrence groups, and Bonferroni correction was performed. U1, maxillary incisor; L1, mandibular incisor; SN, Sella–Nasion; IMPA, incisor mandibular plane angle; T1, before treatment; T2, after treatment; Δ, T2-T1; ICP, interproximal contact point; ABC, alveolar bone crest; A-P, anteroposterior; NS, not significant; * *p* < 0.05; ** *p* < 0.01; *** *p* < 0.001.

**Table 7:** Comparison of black triangle incidence between the young and adult groups in the maxilla and mandible (means ± SD) ^†^.

	Maxilla			Mandible		
	Young	Adult	Significance	Young	Adult	Significance
Treatment period, month	30.23 ± 12.30	38.43 ± 18.39	*	30.23 ± 12.30	38.43 ± 18.39	*
Tooth shape	1.54 ± 2.08	1.23 ± 0.11	NS	1.28 ± 0.15	1.28 ± 0.19	NS
ΔU1 to SN /ΔIMPA, °	−2.88 ± 10.49	−7.69 ± 10.37	*	−0.21 ± 20.15	−4.30 ± 10.50	NS
Horizontal movement of U1/L1, mm	−0.37 ± 1.15	−0.41 ± 0.62	NS	0.30 ± 1.32	0.14 ± 2.10	NS
Vertical movement of U1/L1, mm	4.38 ± 20.32	−0.21 ± 1.43	NS	−0.08 ± 0.93	−1.97 ± 13.63	NS
Δ Distance from ICP to ABC, mm	−0.60 ± 0.57	−0.56 ± 1.24	NS	0.68 ± 0.52	0.76 ± 1.19	NS
Root angulation at T2, °	5.03 ± 3.37	5.26 ± 2.75	NS	5.47 ± 3.73	5.29 ± 3.41	NS
A-P overlapped distance, mm	0.02 ± 0.06	0.11 ± 0.42	NS	0.09 ± 0.23	0.08 ± 0.27	NS
Transverse overlapped distance, mm	−0.03 ± 10.19	−0.38 ± −0.22	NS	0.00 ± 0.04	0.01 ± 0.048	NS
Rotation, °	−12.78 ± 24.30	−12.31 ± 30.48	NS	−14.33 ± 19.16	−11.58 ± 32.08	NS

^†^ B Independent *t*-tests were used to compare between the young and adult groups, and Bonferroni correction was performed. U1, maxillary incisor; L1, mandibular incisor; SN, Sella–Nasion; IMPA, incisor mandibular plane angle; T1, before treatment; T2, after treatment; Δ, T2-T1; ICP, interproximal contact point; ABC, alveolar bone crest; A-P, anteroposterior; NS, not significant; * *p* < 0.05; ** *p* < 0.01; *** *p* < 0.001.

**Table 8:** Comparison of means (±SD) between the extraction and non-extraction groups in the maxilla and mandible ^†^.

	Maxilla			Mandible		
	Extraction	Non-Extraction	Significance	Extraction	Non-Extraction	Significance
Treatment period, month	35.98 ± 15.80	34.34 ± 17.51	NS	36.98 ± 18.26	33.40 ± 17.80	NS
Age, y	1.51 ± 1.90	1.22 ± 0.08	NS	1.28 ± 1.15	1.27 ± 0.10	NS
Tooth shape	−9.77 ± 11.13	−1.99 ± 8.67	***	−5.35 ± 18.53	−0.99 ± 11.04	NS
ΔU1 to SN /ΔIMPA, °	−0.51 ± 0.59	−0.28 ± 1.62	NS	0.12 ± 1.37	0.27 ± 2.18	NS
Horizontal movement of U1/L1, mm	1.85 ± 13.20	1.42 ± 13.00	NS	−0.13 ± 0.58	−2.21 ± 14.70	NS
Vertical movement of U1/L1, mm	−0.38 ± 0.87	−0.76 ± 1.13	NS	−0.90 ± 1.08	−0.55 ± 0.83	NS
Δ Distance from ICP to ABC, mm	5.39 ± 3.17	4.95 ± 2.85	NS	5.43 ± 3.13	5.28 ± 3.90	NS
Root angulation at T2, °	0.09 ± 0.44	0.05 ± 0.17	NS	0.10 ± 0.32	0.06 ± 0.15	NS
A-P overlapped distance, mm	−0.04 ± 0.24	−0.03 ± −0.17	NS	0.008 ± 0.04	0.006 ± −0.04	NS
Transverse overlapped distance, mm	−16.14 ± 34.13	−9.051 ± 20.51	NS	−18.13 ± 29.18	−7.56 ± 25.12	NS
Rotation, °	35.98 ± 15.80	34.34 ± 17.51	NS	36.98 ± 18.26	33.40 ± 17.80	NS

^†^ B Independent *t*-tests were used to compare between the extraction and non-extraction groups, and Bonferroni correction was performed. U1, maxillary incisor; L1, mandibular incisor; SN, Sella–Nasion; IMPA, incisor mandibular plane angle; T1, before treatment; T2, after treatment; Δ, T2-T1; ICP, interproximal contact point; ABC, alveolar bone crest; A-P, anteroposterior; NS, not significant; * *p* < 0.05; ** *p* < 0.01; *** *p* < 0.001.

**Table 9:** Relationship between severity of black triangle and parameters related to treatment by simple logistic regression analysis ^†^.

	Maxilla			Mandible		
	B	SE	Significance	B	SE	Significance
Treatment period, month	0.045	0.016	**	0.026	0.015	NS
Age, y	0.189	0.044	***	0.180	0.045	***
Tooth shape	−0.200	−0.289	NS	0.052	1.602	NS
ΔU1 to SN /ΔIMPA, °	−0.070	0.022	**	0.006	0.014	NS
Horizontal movement of U1/L1, mm	−0.166	0.243	NS	−0.079	0.124	NS
Vertical movement of U1/L1, mm	0.062	0.203	NS	−0.235	0.316	NS
Δ Distance from ICP to ABC, mm	−0.138	0.208	NS	0.049	0.260	NS
Root angulation at T2, °	0.027	0.048	NS	−0.058	0.047	NS
A-P overlapped distance, mm	0.697	0.891	NS	0.725	1.125	NS
Transverse overlapped distance, mm	−0.698	1.083	NS	6.914	5.014	NS
Rotation, °	0.003	0.007	NS	−0.002	0.008	NS

^†^ B Independent *t*-tests were used to compare between the extraction and non-extraction groups, and Bonferroni correction was performed. U1, maxillary incisor; L1, mandibular incisor; SN, Sella–Nasion; IMPA, incisor mandibular plane angle; T1, before treatment; T2, after treatment; Δ, T2-T1; ICP, interproximal contact point; ABC, alveolar bone crest; A-P, anteroposterior; NS, not significant; * *p* < 0.05; ** *p* < 0.01; *** *p* < 0.001.

**Table 10:** Main contributing factors to black triangle in the maxilla by multiple logistic regression analysis ^†^.

	B	SE	Significance	OR
Treatment period, month	0.030	0.018	NS	1.030
Age, y	0.172	0.044	***	1.087
Δ U1 to SN	−0.062	0.027	*	0.940

^†^ Multiple logistic regression analysis was used. B indicates beta coefficient; SE, standard error; OR, odds ratio; U1, maxillary incisor; SN, Sella–Nasion; Δ, T2-T1; L1, mandibular incisor; NS, not significant; * *p* < 0.05; ** *p* < 0.01; *** *p* < 0.001.

## Data Availability

The data presented in this study are available upon request to the corresponding author due to the protection of patient privacy.
